# Comparative analysis of human gut- and blood-derived mononuclear cells: contrasts in function and phenotype

**DOI:** 10.3389/fimmu.2024.1336480

**Published:** 2024-02-20

**Authors:** Stephanie C. Burke Schinkel, Priscila O. Barros, Tamara Berthoud, Siddappa N. Byrareddy, Michaeline McGuinty, D. William Cameron, Jonathan B. Angel

**Affiliations:** ^1^ Chronic Diseases Program, Ottawa Hospital Research Institute, Ottawa, ON, Canada; ^2^ Department of Pharmacology and Experimental Neuroscience, College of Medicine, University of Nebraska Medical Center, Omaha, NE, United States; ^3^ Department of Medicine, Division of Infectious Diseases, The Ottawa Hospital, University of Ottawa, Ottawa, ON, Canada; ^4^ Department of Biochemistry, Microbiology and Immunology, University of Ottawa, Ottawa, ON, Canada

**Keywords:** human, T cell, mucosa, cell trafficking, cytokines

## Abstract

**Introduction:**

Alterations in the gut immune system have been implicated in various diseases.The challenge of obtaining gut tissues from healthy individuals, commonly performed via surgical explants, has limited the number of studies describing the phenotype and function of gut-derived immune cells in health.

**Methods:**

Here, by means of recto-sigmoid colon biopsies obtained during routine care (colon cancer screening in healthy adults), the phenotype and function of immune cells present in the gut were described and compared to those found in blood.

**Results:**

The proportion of CD4^+^, CD8^+^, MAIT, γδ+ T, and NK cells phenotype, expression of integrins, and ability to produce cytokine in response to stimulation with PMA and ionomycin. T cells in the gut were found to predominantly have a memory phenotype as compared to T cells in blood where a naïve phenotype predominates. Recto-sigmoid mononuclear cells also had higher PD-1 and Ki67 expression. Furthermore, integrin expression and cytokine production varied by cell type and location in blood vs. gut.

**Discussion:**

These findings demonstrate the differences in functionality of these cells when compared to their blood counterparts and validate previous studies on phenotype within gut-derived immune cells in humans (where cells have been obtained through surgical means). This study suggests that recto-sigmoid biopsies collected during colonoscopy can be a reliable yet more accessible sampling method for follow up of alterations of gut derived immune cells in clinical settings.

## Introduction

Given its association with an ever-growing list of heterogeneous disorders, an increased interest in the immune elements of the gastrointestinal tract has emerged. Alterations in the gut are seen or suspected in conditions with different etiologies; e.g. autoimmune [Type 1 Diabetes, Multiple Sclerosis and inflammatory bowel disease (IBD)], diet and lifestyle (alcoholic liver disease, obesity), and chronic infections [Human Immunodeficiency Virus (HIV), Hepatitis C Virus (HCV)] (1–9). The close interaction between external stimuli, gut-associated lymphoid tissue (GALT), and blood vessels underlie numerous potential relationships that could contribute to the pathophysiology of these diseases. Such alterations, be they a cause or result of disease, include increased intestinal permeability and inflammation, which can lead to tissue damage both in the intestine and elsewhere. While numerous studies have evaluated gut-derived immune cells in the above diseases, heterogeneous sampling techniques make it difficult to extrapolate results to other settings and make replication a challenge. In addition, evaluation of how gut-derived immune cells in the condition differs from that of healthy individuals is often not possible within the constraints of the study. This study aims to use the standard of care technique of recto-sigmoid colon biopsy to sample gut-derived immune cells and compare them to those in blood.

Cells that migrate into the gut differ from those that remain in the blood by their surface expression of key markers. Integrin expression on activated lymphocytes enables them to interact with ligands present in the high endothelial venules of the GALT, then migrate into the lamina propria. For instance, heterodimers alpha4beta7 (α4β7) and αEβ7 (CD103 E Integrin) bind to mucosal cell adhesion molecule-1 (MAdCAM-1) and E-cadherin, respectively (10–12), and are implicated in Crohn’s disease and ulcerative colitis (13) and in HIV pathogenesis (14, 15). Both chemokine/ligand axes CCR9/CCL25 and CCR6/CCL20 also play roles, with CCR6 being critical for migration into Peyer’s patches and the small intestine (11, 16–18).

Previous studies have compared the proportions of cell populations derived from different locations in the gut. For instance, evaluation of intestinal lymphoid follicles in the sub-mucosa and mucosa of the ileum, along the length of the colon, and in non-GALT lamina propria found differing proportions of B cells and subtypes of T cells, along with memory subsets that were predominately central or effector memory depending on the location (19). Ickler et al. evaluated mononuclear cells in the lamina propria after removal of epithelium, finding over half are CD4^+^ T cells (58.3% ± 26.4%), followed by CD8^+^ T cells (21.2% ± 15.77%), B cells (9.8% ± 18.10%), and NK cells (2.2% ± 2.0) (20). These studies are; however, examples of ones using abdominal surgical explants and did not compare their findings to cell populations in peripheral blood.

Chemokine receptors and integrins that have an important function in the GALT can also affect migration into other tissues, potentially contributing to diseases outside the gut. CCR5 is involved in numerous diseases; CCR5^+^ cells are found in the CNS in MS and other conditions with CNS inflammation, CCR5 ligands are found in synovial fluid during rheumatoid arthritis (21), and CCR5 is an HIV-binding co-receptor (20, 22). CCR6 is also a CNS-homing chemokine receptor and enables cell migration to the CNS by means of the choroid plexus constitutive CCL20 expression (23–25). α4β7 is expressed on activated CD4^+^ T cells and can bind to HIV gp120 independent of CD4 binding (10, 26) and facilitate cell-to-cell spread (14). As these integrins and chemokine receptors regulate cell migration, they may play a role in both disease progression and represent a therapeutic target for controlling diseases that involve the GALT.

While access to large samples of abdominal explants and stereomicroscopic dissection of the various layers of intestinal samples is valuable in the detailed evaluation of very specific diseases, a simple, easily accessible, and replicable sampling method is needed to evaluate healthy gut-derived immune cells. Herein, using standard of care recto-sigmoid colon biopsy in heathy individuals undergoing colon cancer screening, we have been able to characterize immune cells present in the colon, as well as their cell surface protein expression and functionality, and compare this to their counterparts in the blood. As we show, the proportions of gut derived immune cells do differ from those in the blood, most significantly in their memory phenotype, expression of integrins CCR5, CCR6, α4β7, and CD103, production of cytokine in response to stimulation, and expression of PD-1. Our data suggest that studying blood alone is insufficient to form conclusions about the effect of disease on the immune system within the intestine and vice versa. With increased insight into how gut-derived immune cells diverge from those in the blood, a better understanding of the impact that different diseases have on the gut will be possible and therefore, measuring these changes in gut tissues are critical to understand the disease pathogenesis and to develop therapeutics.

## Materials and methods

### Sample collection and cell isolation

Blood and recto-sigmoid colon biopsies were collected from un-matched healthy individuals who provided written informed consent, as per study approval by the Ottawa Hospital Research Institute Ethics Board (OHRI REB 2005256-01H) (Ottawa, ON, Canada). Peripheral blood mononuclear cells (PBMC) were isolated from heparinized whole blood samples via density centrifugation. Briefly, blood was layered on Lymphoprep (Stemcell, Vancouver BC), centrifuged, and PBMC were collected and washed in PBS. PBMC were cultured in RPMI-1640 with 10% FBS, 2mM L-glutamine, and 1% Penicillin/Streptomycin (Gibco).

**Figure 1:**
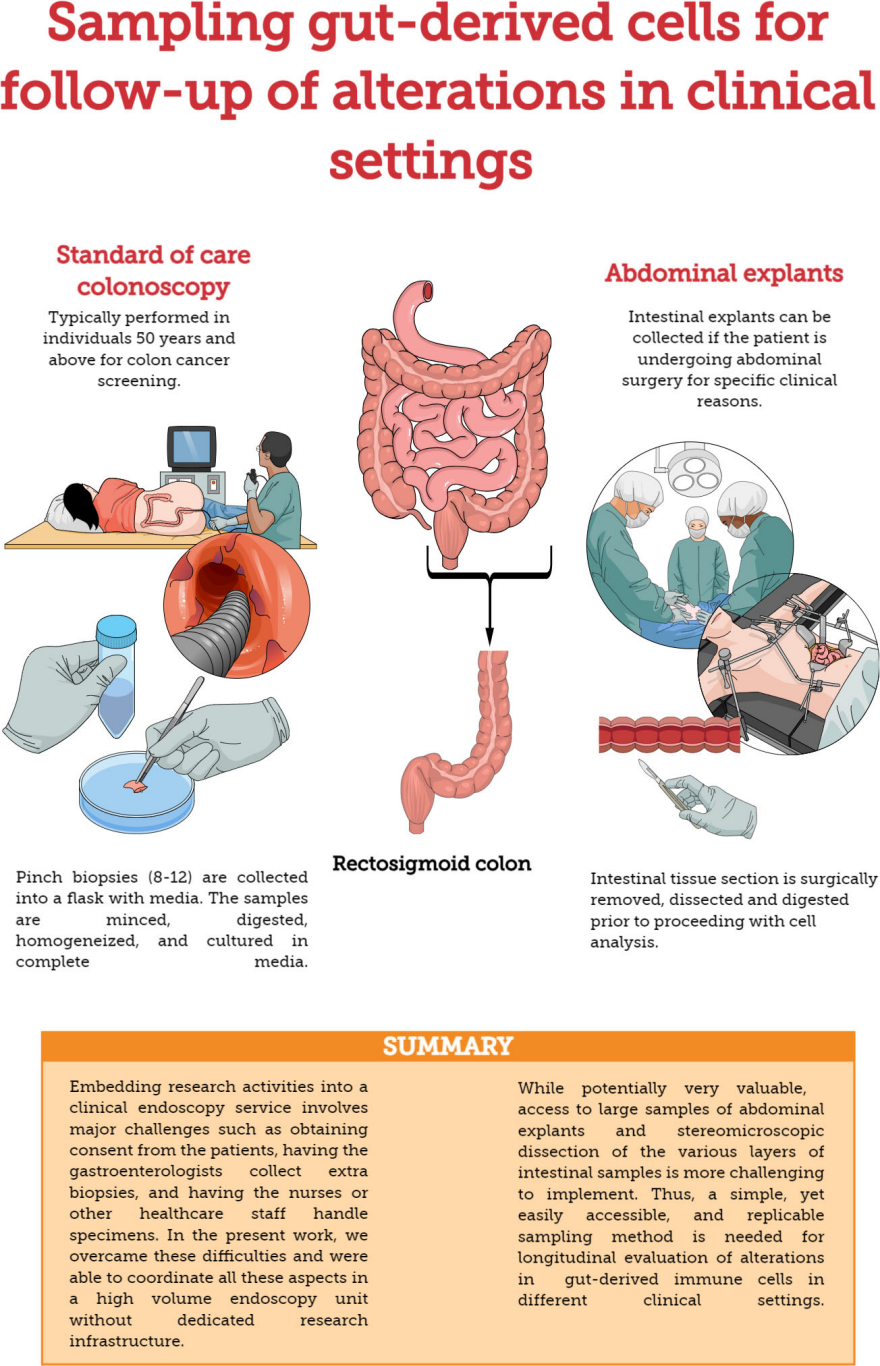
Methods for sampling gut-derived cells for studies of gut immunology. Comparison of standard of care colonoscopy and abdominal explants for evaluating/monitoring gut immune function.

Recto-sigmoid mononuclear cells (RMC) were isolated from pinch biopsies collected from individuals undergoing routine screening colonoscopies and without any visible pathology or known disease (**Figure 1**). Pinch biopsies were taken from 8-12 locations in the recto-sigmoid colon. Cells were isolated from the collected tissue via enzyme digestion and mechanical dissociation. Briefly, biopsy tissue was collected into and manipulated in RPMI supplemented with 10% FBS, 1% HEPES, 1% Penicillin/Streptomycin (Gibco), and Gentamicin (0.1mg/ml, Sigma) (RMC media). Tissue was cut into small pieces and digested in RMC media with Collagenase Type IV (1000U, Gibco), DNase1 (10U, Sigma) and Tazocin (2.3mg Piperacillan, 0.29g Tazobactam, Sandoz, Basel, Switzerland) for 2 hours at 37°C and 250rpm. Tissue was then needle homogenized with 18 then 20 gauge needles, filtered through 40uM filter, washed and cultured in RMC media.

### Flow cytometry

To evaluate cell surface markers, isolated cells were stained with LIVE/DEAD™ Fixable Aqua Dead Cell Stain Kit (Life Technologies) for 25min at room temperature in PBS, washed in 2% FBS PBS, then incubated with appropriate antibodies for 30min at 4°C (see **Table 1**). Cells were fixed and permeabilized using the True-Nuclear™ Transcription Factor Buffer Set (Biolegend); cells were incubated in the fix solution for 45min at room temperature, then washed twice in the wash buffer. A subset of cells was then stained with Ki67 for 30min at room temperature, washed again in wash buffer, then a final wash in 2% FBS PBS and resuspended in PBS to be analyzed via flow cytometry.

**Table 1:** Flow cytometry antibodies used for phenotype panel.

Antibody	Company	Catalog #
APC/Cy7 anti-human **CD45** Antibody clone 2D1	Biolegend	368516
Brilliant Violet 650™ anti-human **CD4** Antibody clone OKT4	Biolegend	317436
**α4β7** APC	Non-Human Primate Reagent Program	
LIVE/DEAD™ Fixable Aqua Dead Cell Stain Kit, for 405 nm excitation	Life Technologies	L34957
Brilliant Violet 785™ anti-human **CD3** Antibody clone UCHT1	Biolegend	300472
Alexa Fluor^®^ 700 anti-human **CD8** Antibody clone SK1	Biolegend	344724
PE/Cy5 anti-human **CD56** (NCAM) Antibody clone MEM-188	Biolegend	304608
Brilliant Violet 711™ anti-human **CD103** (Integrin αE) Antibody clone Ber-ACT8	Biolegend	350222
Brilliant Violet 605™ anti-human **CD16** Antibody clone 3G8	Biolegend	302040
PerCP/Cy5.5 anti-human CD185 (**CXCR5**) Antibody clone J252D4	Biolegend	356910
PE-Cy™7 Mouse Anti-Human **CD45RA** Clone HI100	BD Biosciences	560675
HUMAN **CCR7** PHYCOERYTHRIN MAB (CLONE 150503)	R&D Systems	FAB197P-100
PE/Dazzle™ 594 anti-human CD279 (**PD-1**) Antibody clone EH12.2H7	Biolegend	329940
Alexa Fluor^®^ 488 anti-human CD196 (**CCR6**) Antibody, clone G034E3	Biolegend	353414
Brilliant Violet 421™ anti-human **Ki-67** Antibody	Biolegend	350506

The values in bold are all cell markers extensively studied and widely known.

To measure intracellular cytokine expression, cells were stimulated with PMA (0.081 µM) and Ionomycin (1.34µM) (500x Cell activation cocktail, Biolegend), Brefeldin A (10ug/ml, Sigma), and GolgiStop (0.5uL/mL, BD Biosciences) for 5 hours. Cells were stained with LIVE/DEAD stain kit for 25min in PBS, washed in 2%FBS PBS, then incubated with appropriate antibodies for 30min at 4°C (see **Table 2**). Cells were fixed for 20min at 4°C and permeabilized by washing with the BD Cytofix/Cytoperm kit (BD). Following intracellular staining for 30 min at 4°C, cells were washed, resuspended in PBS, and finally analyzed via flow cytometry.

**Table 2:** Flow cytometry antibodies used for intracellular cytokine stimulation.

Antibody	Company	Catalog #
APC/Cy7 anti-human **CD45** Antibody clone 2D1	Biolegend	368516
Brilliant Violet 650™ anti-human **CD4** Antibody clone OKT4	Biolegend	317436
**α4β7** APC	Non-Human Primate Reagent Program	
LIVE/DEAD™ Fixable Aqua Dead Cell Stain Kit, for 405 nm excitation	Life Technologies	L34957
Alexa Fluor^®^ 700 anti-human **CD3** Antibody clone UCHT1	Biolegend	300424
Brilliant Violet 711™ anti-human **CD8** Antibody clone SK1	Biolegend	344734
PE/Cy7 anti-human **CD161** Antibody clone HP-3G10	Biolegend	339918
Alexa Fluor^®^ 488 anti-human CD195 (**CCR5**) Antibody clone J418F1	Biolegend	359104
Brilliant Violet 605™ anti-human **TCR Vα7.2** Antibody clone 3C10 (100 tests)	Biolegend	351720
PE/Dazzle™ 594 anti-human **TCR γ/δ** Antibody clone B1 (100 tests)	Biolegend	331226
PerCP/Cy5.5 anti-human **IL-22** Antibody clone 2G12A41	Biolegend	366710
PE, **IL-17A** Monoclonal Antibody (eBio64DEC17)	eBiosciences	12-7179-42
Pacific Blue™ anti-human **IFN-γ** Antibody clone 4S.B3	Biolegend	502522
Brilliant Violet 785™ anti-human **TNF-α** Antibody clone MAb11	Biolegend	502948

The values in bold are all cell markers extensively studied and widely known.

Antibodies were obtained from Biolegend, Life Technologies, the NIH’s Non-Human Primate Reagent Program, eBiosciences, Cedarlane, and Becton Dickinson. The Anti-alpha4/beta7 [A4B7R1]-APC antibody was engineered and produced by the Nonhuman Primate Reagent Resource (NIH Nonhuman Primate Reagent Resource Cat#PR-1421,RRID:AB_2819257). Compensation beads (Anti-Mouse Ig, κ/Negative Control Compensation Particles Set, BD or ArC™ Amine Reactive Compensation Bead Kit, Life Technologies) or PBMCs were used as compensation controls and PBMC were used for negative staining and FMO controls. Samples were analyzed on a Becton Dickinson Fortessa LSR running FACS Diva software 8.0 (BD Biosciences, San Jose, USA).

### Data analysis

Flow cytometry data were analysed using FlowJo v10 (Tree Star, Ashland, OR). Positive gates were set by FMO or negative staining controls. Graphs and statistics were done using GraphPad Prism 8 (GraphPad Software, Inc). Unpaired student’s t-tests were performed. Data are presented as mean ± SEM. All tests were two-tailed, and differences were considered statistically significant when p<0.05.

For the tSNE analysis, FlowJo Downsample plugin was used to ensure similar number of events in all samples. Flow Cytometry Standard 3.0 (FCS 3.0) files from PBMC and RMC were pooled together, using the concatenation function in FlowJo software, to obtain a single file. Keywords for each sample compartment (PBMC or RMC) were applied to enable posterior gating and comparison. FlowJo native tSNE function was run on the concatenated file, at the following parameters: t-SNE dimensions=2, Nearest neighbors=Approximate, Perplexity=20.0, and Maximum iterations=3000, Algorithm: FItSNE (27). FlowJo FlowSOM plugin was run for 24 clusters (28) and, finally, FlowJo ClusterExplorer plugin was used for generating the graphs with tSNE and FlowSOM data.

## Results

### Immune cell phenotype differs depending on their location

**Figure 2:**
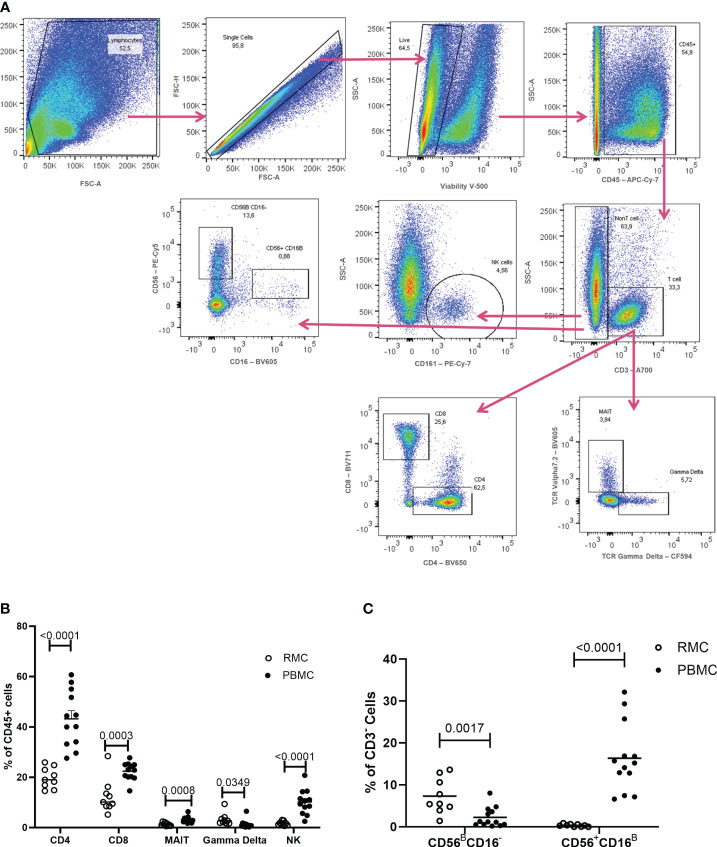
Flow Cytometry gating strategy and cell proportions in blood and recto-sigmoid colon derived mononuclear cells. **(A)** Gating strategy for distinguishing the different cell populations as measured by flow cytometry, shown in a rectosigmoid colon biopsy sample. The proportions of CD4^+^ T cells, CD8^+^ T cells, MAIT, gd T cells, and NK cells **(B)** and of NK cell sub-populations **(C)**, within CD45+ cells, in RMC (n=9) and PBMC (n=13) are demonstrated (unpaired student’s t-tests, data are presented as mean ± SEM).

To first characterize phenotypic differences between immune cells present in the blood and in the gut, recto-sigmoid mononuclear cells (RMC) were isolated from recto-sigmoid pinch biopsies collected from healthy individuals undergoing routine screening colonoscopy. Participants had a mean age of 65.7 ± 8.5 years (7 male: 2 female). PBMC were isolated from non-matched donors with an average age of 39.2 ± 9.8 years (6 male: 7 female). RMC and PBMC were identified as live, single cell, and CD45^+^ (**Figure 2A**). Of cells expressing CD45, all the cell populations studied here have significantly different proportions when comparing blood and recto-sigmoid colon. CD4^+^ T cells form the largest component in both recto-sigmoid colon (19.8 ± 4.1%) and blood (43.2 ± 11.14%) (p<0.0001), followed by CD8^+^ T cells, (12.5 ± 6.8% and 22.4 ± 3.5%, respectively) (p=0.0003), and MAIT cells (TCRVα7.2^+^) (1.3 ± 0.7 and 3.2 ± 1.3%) (p=0.0008). Gamma delta (γδ)^+^ T cells also differ significantly, with a greater proportion in recto-sigmoid colon (3.3 ± 2.4%) compared to the peripheral blood (1.3 ± 1.7%, p=0.0349). Natural killer cells are also found in different proportions, with significantly more NK cells (CD3^-^CD161^+^) in the blood (10.14 ± 4.9%) than recto-sigmoid colon (1.7 ± 0.8%, p<0.0001) (**Figure 2B**). When looking at NK cell subsets, among CD3- cells, CD56^Bright^CD16^-^ cells are found to a greater extent in the recto-sigmoid colon (7.32 ± 1.4% vs 2.27 ± 0.65% in blood; p=0.0017) while CD56^+^CD16^Bright^ are more prevalent in the peripheral blood (16.36 ± 2.3% vs 0.35 ± 0.1% in recto-sigmoid colon; p<0.0001) (**Figure 2C**).

**Figure 3:**
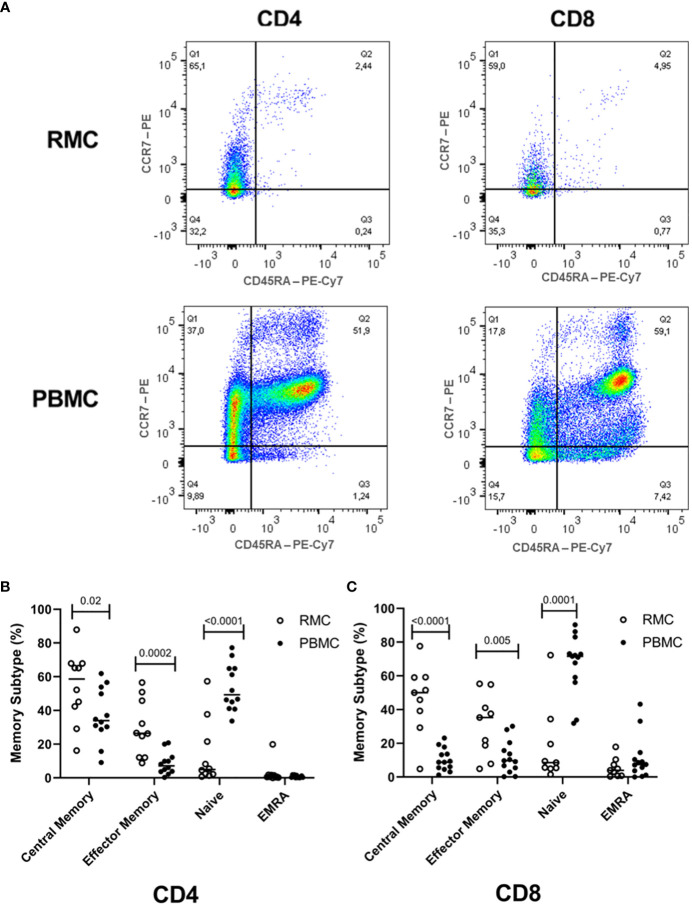
Memory phenotype of CD4^+^ and CD8^+^ T cells. **(A)** Representative dot plot RMC (n=9) and PBMC (n=13) CD4^+^ T cells and CD8^+^ T cells as measured by flow cytometry. **(B)** Memory subtype of CD4^+^ T cells and **(C)** memory subtype of CD8^+^ T cells. Central Memory (CD45RA-CCR7^+^), Effector Memory (CD45RA-CCR7-), Naïve (CD45RA+CCR7^+^), Effector Memory Re-expressing CD45RA – EMRA (CD45RA+CCR7^-^) subtypes are demonstrated (unpaired student’s t-tests, data are presented as mean ± SEM).

While the overall proportions of CD4^+^ and CD8^+^ T cells are similar in blood and recto-sigmoid colon, their memory phenotype differs significantly. Naïve cells (CD45RA^+^CCR7^+^) form the majority of cells in the blood (54.1 ± 3.7% for CD4^+^, 66.8 ± 4.9% for CD8^+^). Still, central memory cells (CD45RA^-^CCR7^+^) represent most T cells in the recto-sigmoid colon (56.7 ± 6.8% for CD4^+^, 46.3 ± 6.9% for CD8^+^), with effector memory (CD45RA^-^CCR7^-^) cells also higher in RMC than PBMC for both T cell types (**Figure 3**).

**Figure 4:**
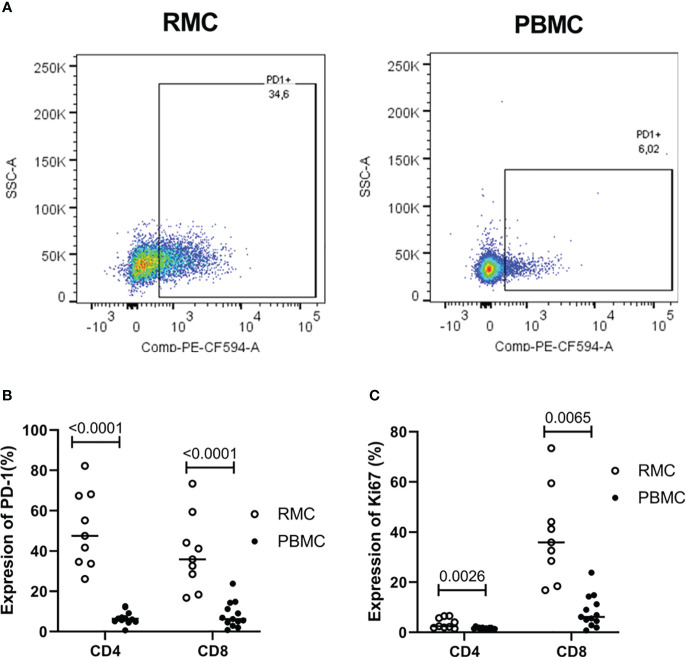
Expression of PD-1 and Ki67 on CD4^+^ and CD8^+^ T cells is higher in rectosigmoid colon-derived RMC than in blood-derived PBMC. **(A)** Representative dot plot of PD1 expression in RMC (n=9) and PBMC (n=13) as measured by flow cytometry. **(B)** Expression of PD-1 and **(C)** Ki67 of CD4^+^ and CD8^+^ T cells (unpaired student’s t-tests, data are presented as mean ± SEM).

As a cell regulator, signaling through PD-1 can control both tolerance and the magnitude of response to stimuli, in addition to being a traditional marker of cell exhaustion [reviewed in (29)]. Expression of PD-1 (**Figure 4A**) was found to be higher on recto-sigmoid colon-derived CD4^+^ and CD8^+^ T cells (50.72 ± 6.23% and 38.92 ± 6.13%, respectively) compared to blood-derived cells (CD4^+^: 6.72 ± 0.87% and CD8^+^: 8.22 ± 1.78%) (p<0.0001 for each comparison of CD4^+^ and CD8^+^ cells) (**Figure 4B**) and was highest on memory (CD45RA^-^) cells (data not shown). The presence of Ki67 in the nucleus indicates that the cell is preparing to divide and is a cell proliferation marker. Ki67 expression was generally lower in CD4^+^ T cells, but statistically higher in recto-sigmoid colon than in blood (3.63 ± 0.70 and 1.58 ± 0.12, respectively; p=0.0026). Ki67 expression in CD8^+^ T cells was also significantly higher in the recto-sigmoid colon than in blood (4.96 ± 1.65% and 1.10 ± 0.28%, respectively; p=0.0065) (**Figure 4C**).

### Chemokine receptor and integrin expression of RMC differ from PBMC

**Figure 5:**
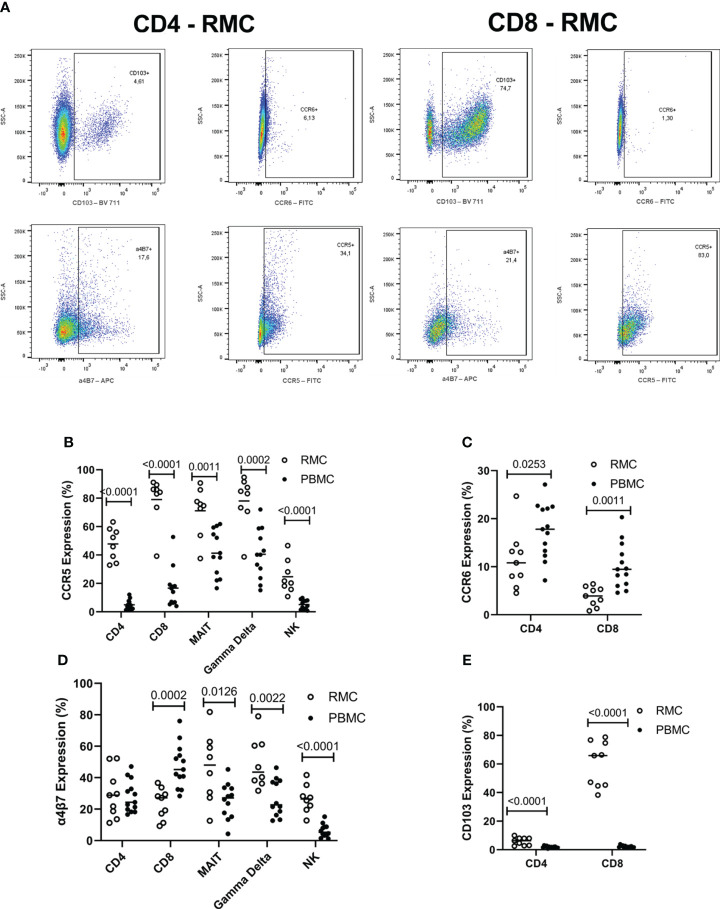
The expression of integrins differs by location and cell subsets. **(A)** Representative dot plot of CD103, CCR6, α4β7 and CCR5 expression in RMC (n=9) and PBMC (n=13) as measured by flow cytometry **(B)** CCR5 expression is highest in RMC than in PBMC. **(C)** CCR6 expression is higher in PBMC than in RMC. **(D)** Expression of α4β7 depends on location. **(E)** CD103 expression is higher in RMC than in PBMC (unpaired student’s t-tests, data are presented as mean ± SEM).

The surface expression of chemokine receptors and integrins important for migration to the GALT was evaluated by flow cytometry on the various cell types (**Figure 5A**). CCR5 promotes leukocyte trafficking, and in doing so promotes clearance of infections [reviewed in (21)]. Across all cell types evaluated, CCR5 expression was higher in the recto-sigmoid colon than in blood. CD8^+^ and yδ T cells express the highest levels of CCR5 in RMC (79.1 ± 6% and 78.1 ± 6.3% respectively), followed by MAIT cells (71.1 ± 6.1%), CD4^+^ T cells (47.8 ± 4%), and lastly NK cells (24.2 ± 4.1%) (**Figure 5B**). The CCR6/CCL20 axis is responsible for cell trafficking to Payers patches and the small intestine (16). Still, it is also a CNS-homing chemokine receptor through the constitutive expression of CCL20 of the choroid plexus and has been identified as a target of interest in multiple sclerosis (23–25). The expression of CCR6 was lower in recto-sigmoid CD4^+^ and CD8^+^ T cells compared to those in the blood (**Figure 5C**), with the highest expression in memory (CD45RA-) cells in both PBMC and RMC (data not shown). The integrin α4β7 binds to MAdCAM-1 (10) present in the high endothelial venules (HEV) of the GALT and plays an important role in capturing immune cells and directing them to the GALT (11). Expression of α4β7 is significantly higher in recto-sigmoid compared to blood-derived MAIT, γδ T and NK cells, while CD4^+^ T cells show no difference, and α4β7 expression on CD8^+^ T cell is higher in blood (**Figure 5D**). Of blood-derived cells, α4β7 expression on CD45RA^+^ CD4^+^ and CD8^+^ T cells is higher than on memory (CD45RA-) cells (data not shown). Integrin αE (CD103) is involved in the lymphocyte-to-epithelial cell-to-cell interaction by binding to E-cadherin and E-cadherin is expressed on most types of endothelial cells, allowing cells to embed in the epithelial layer to become intraepithelial lymphocytes (12). CD103 has the highest expression on CD8^+^ T cells from the recto-sigmoid colon, with both CD4^+^ and CD8^+^ T cells in RMC expressing significantly more CD103 than in PBMC (**Figure 5E**).

### The function of PBMC vs. RMC

**Figure 6:**
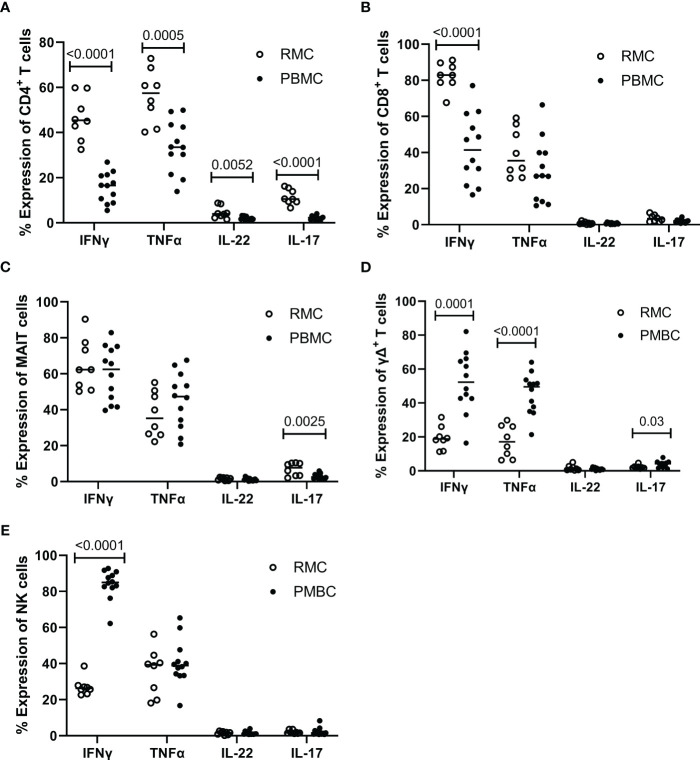
Expression of intracellular cytokines in response to stimulation. Cells from RMC (n=8) and PBMC (n=12) were stimulated with PMA and Ionomycin in the presence of golgi stop and brefeldin A for 5 hours and their intracellular expression of cytokine was measured by flow cytometry, relative to unstimulated (fold change). **(A)** CD4^+^ T cells **(B)** CD8^+^ T cells **(C)** MAIT cells **(D)** γδ T cells **(E)** NK cells (unpaired student’s t-tests, data are presented as mean ± SEM).

**Figure 7:**
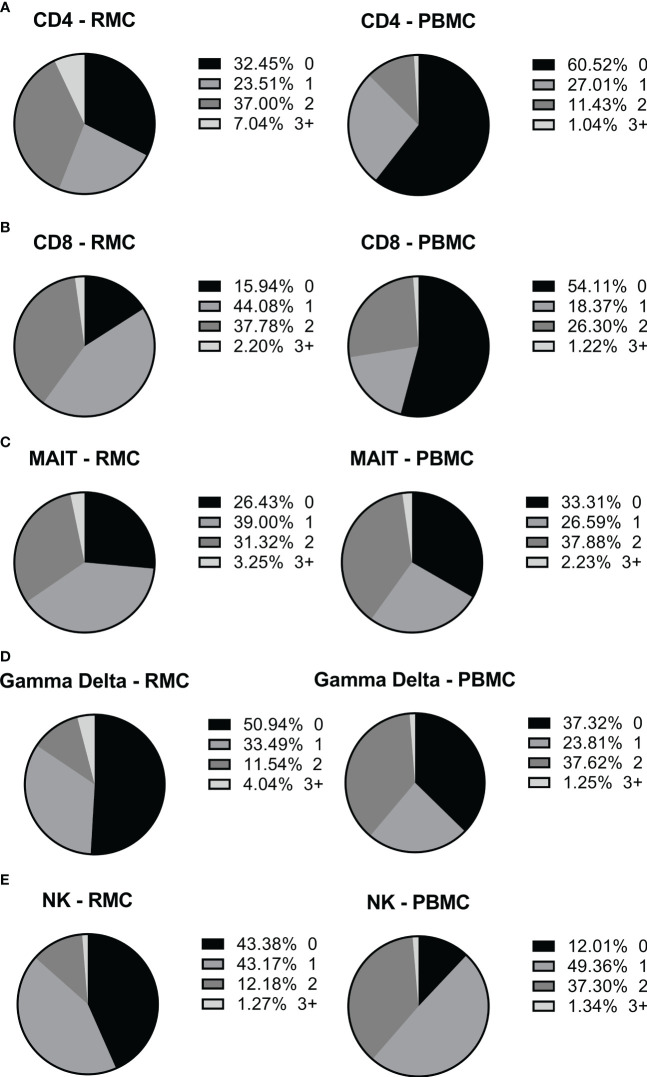
The ability of different cells to express more than one intracellular cytokine in response to stimulation depends on cell type and location. The expression of multiple cytokines in response to stimulation by the same cell, in PBMC (n=12) and RMC (n=8), was determined by Boolean gating. **(A)** CD4^+^ T cells **(B)** CD8^+^ T cells **(C)** MAIT cells **(D)** γδ T cells **(E)** NK cells.

Determining how cells present in the gut respond to stimuli can provide insight into their function and indicate if they perform differently than those in the blood. To assess the functionality of isolated cells through their response to activation, bulk PBMC and RMC were stimulated with PMA and Ionomycin for 5 hours in the presence of brefeldin A and golgi stop, and the production of IFNγ, TNFα, IL-17 and IL-22 was evaluated via flow cytometry. For all cytokines measured, CD4^+^ T RMC produced higher levels in response to stimulation when compared to CD4^+^ T PBMC (**Figure 6A**). Further, RMC were also more polyfunctional (more cells expressing greater than one cytokine) and had fewer cells expressing no cytokines than PBMC (**Figure 7A**). In CD8^+^ T cells, RMC produced more IFNγ, and had fewer cells that produced no cytokines, but there was no difference in polyfunctionality (**Figures 6B**, **7B**). MAIT RMC had more cells expressing IL-17, with no significant difference in other cytokines and no difference in polyfunctionality (**Figures 6C**, **7C**). Unlike the other T cells, fewer yδ T RMC expressed IFNγ, TNFa and IL-17 compared to γδ T PBMC (**Figure 6D**), and γδ T PBMC had more cells co-expressing 2 cytokines when compared to γδ T in RMC (**Figure 7D**). NK cells had a similar trend, as NK cells in PBMC expressed more IFNy (**Figure 6E**) and had more cells co-expressing 2 cytokines while RMC had more NK cells expressing no cytokines (**Figure 7E**).

**Figure 8:**
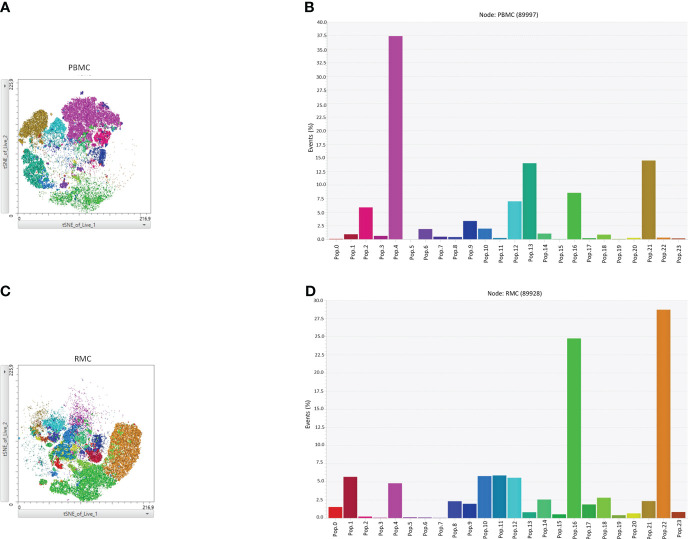
FItSNE projection of the various cytokine-expressing cell subtypes in PBMC and RMC. Data from healthy donor PBMC (n=8) or RMC (n=11) samples, obtained via flow cytometry, were concatenated to generate a single FItSNE projection of the different unbiased cell clusters based on their cytokine and markers expression, calculated with FlowSOM. The FItSNE projection along with the percentage of events of each cell cluster for both PBMC **(A, B)** and RMC **(C, D)** are shown. FItSNE analysis was performed with the tSNE function in FlowJo, FItSNE algorithm, with 3000 iterations, and a perplexity of 20.0.

**Figure 9:**
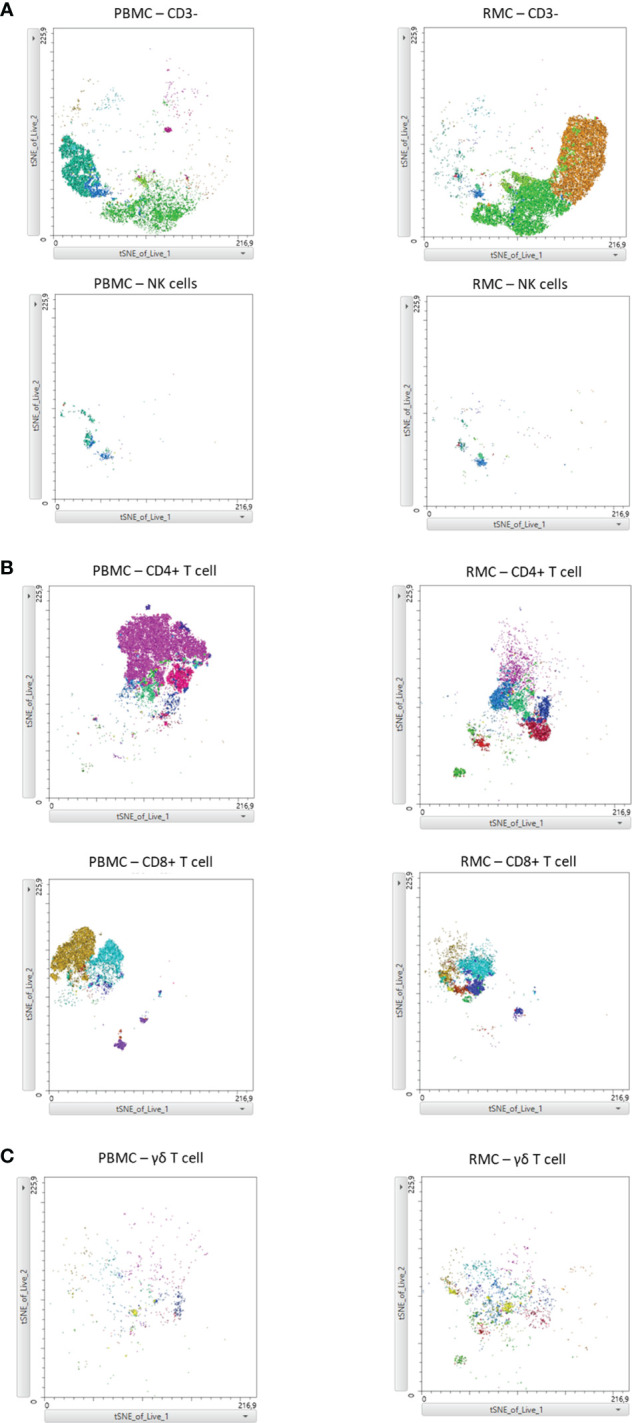
FItSNE projection of the main cell groups among the various cytokine-expressing cell subtypes in PBMC and RMC. Layers of different cell populations were removed from the original tSNE projection for better visualization of the main cell groups in PBMC (n=8) and RMC (n=11); NK and other CD3- cells **(A)**, CD4^+^ and CD8^+^ T cells **(B)**, and γδ T cells are demonstrated **(C)**. FItSNE analysis was performed with the tSNE function in FlowJo, FItSNE algorithm, with 3000 iterations, and a perplexity of 20.0.

Finally, we generated a FItSNE projection with the intracellular cytokine staining flow cytometry data to better visualize the differences in polyfunctionality of the various cell subsets in the gut and the blood (**Figure 8**). The cell clusters are generated with FlowSOM, an algorithm for unbiased clustering, and it is based on single cell similitude regarding the expression of cytokines and the cell subset markers (28). Each one of the 24 cell clusters (Pop.0 to Pop.23) has different patterns of cytokine (IFN-γ, TNF-α, IL-17, and IL-22) and cell subset marker expression. These markers were all analyzed concomitantly. A FItSNE projection of the cell clusters and the percentage of events of each cell clusters, present only in PBMC (**Figures 8A, B**) and RMC (**Figures 8C, D**) are demonstrated. Layers of different cell populations, based on traditional manual gating (**Figure 2A**), were removed from the original tSNE projection (**Figure 9**) for better visualization of the main cell groups: NK and other CD3- cells (**Figure 9A**), CD4^+^ and CD8^+^ T cells (**Figure 9B**), and γδ T cells (**Figure 9C**). This approach demonstrates visually, with an unbiased technique, the differences in function observed when comparing blood and gut-derived cells.

## Discussion

In this study, the immune cells present in the gut, specifically the recto-sigmoid colon, were evaluated outside of any disease context using a standard biopsy technique. To our knowledge, this is the first study to thoroughly describe cell phenotype and function of various cell subsets in healthy human gut, derived from biopsies collected during screening colonoscopies, and compare it to peripheral blood. The evaluation of these healthy cells provides a better understanding of what observations can be attributed to disease states. The differences in cell proportions, memory phenotype, integrin expression and cytokine production between gut and blood highlight the importance of this.

The first significant finding of this study is that while the proportion of CD3^+^ cells that were CD4^+^, CD8^+^, and TCRvα7.2(MAIT)^+^ were similar in PBMC and RMC, there were more γδ T cells in the gut and more NK cells in the blood. While the proportion of CD4^+^ and CD8^+^ T cells observed in blood and recto-sigmoid colon are similar, their memory profile is considerably different. Blood-derived T cells were predominantly naïve, whereas recto-sigmoid colon-derived T cells are predominantly memory (CD45RA^-^). This difference between the proportion of memory cells in the gut and peripheral blood compartments is probably a result of high antigenic exposure characteristic of the gut environment. This finding is in line with early studies in intestinal lamina propria T cells (30) and with a more recent study that showed that intra-individual comparisons of blood versus gut (rectosigmoid colon) demonstrated higher proportions of memory cells in gut (31).

The proportions of T and NK cells we observed in the recto-sigmoid colon are similar to what has been described in lamina propria by Ickler et al. (20) (exact location within the GI tract unspecified) and in the colon by Fenton et al. (19), both of whom obtained, through open surgical procedures, intestinal explants from which the epithelium was removed and specific layers were sampled. The biopsy method in the present report may have included portions of the intestinal epithelium, lamina propria and intestinal lymphoid follicles of the recto-sigmoid colon [a structure is evaluated in (19)], indicating that the lack of dissection did not appear to impact the identification of cells in the gut.

Our results showing γδ T cell being enriched in the gut are consistent with what has been shown in other tissues including the lung, intestines and skin (32). Interestingly, NK cells are present in a scattered fashion in the epithelial layer of the gut instead of forming lymphoid aggregates like other cell types (33). Despite this, the proportions of NK cells obtained in our study was not influenced by the distinct distribution of cells in the tissue and was consistent between the use of pinch biopsies and previously described tissue explants. As for the proportion of central vs. effector memory T cells, others have shown this varies depending on exact sampling location in the different layers of the intestinal tissues (mucosa, sub-mucosa, intestinal lymphoid follicle, lamina propria, etc.) (19, 34, 35). Our results most closely mimic those found in lamina propria of the colon (19).

Higher expression of PD-1 was observed on cells with a memory phenotype (CD45RA-) and expression was higher in recto-sigmoid CD4^+^ and CD8^+^ T cells, while Ki67 was high in RMC CD8^+^ T cells, but low in RMC and PBMC-derived CD4^+^ T. The increased Ki67 found here in rectal CD8^+^ T cells is consistent with other studies that found higher Ki67 expression in CD8^+^ T cells in mucosa than in blood (31, 36). The low overall Ki67 expression in CD4^+^ T cells is also consistent with other works and suggests that persistence of CD4^+^ T cells in the tissue is not due to proliferation but longevity (37).

Evaluation of integrin expression on PBMC and RMC revealed that while typically higher in RMC, integrin expression levels depended on cell type. CCR5 expression on all gut-derived cells was higher than their blood-derived counterparts, but CCR6 expression on CD4^+^ and CD8^+^ T cells was higher in PBMC than in RMC, similar to a previous study (38). Further, when compared to PBMC, the recto-sigmoid expression of α4β7 was higher in γδ, MAIT and NK cells, but lower in CD8^+^ T cells, with no difference in CD4^+^ T cells. The final integrin evaluated, CD103, had the highest expression on RMC CD8^+^ T cells with low levels of expression on CD4^+^ T cells in both locations. The high expression of CCR5 on all gut-derived cells has been previously demonstrated on lymphocytes in intestinal lamina propria [reviewed in (21)]. The low expression of CD103, except in recto-sigmoid CD8^+^ T cells, is expected as few circulating peripheral lymphocytes express the integrin (36, 39, 40), and CD8^+^ T cells have been shown to express more CD103 than CD4^+^ T cells in many tissue types including lymph nodes and the intestines (37, 40).

All cell types evaluated within PBMC and RMC produced appreciable amounts of IFNγ and TNFα in response to stimulation. Compared to blood-derived cells, higher proportions of cytokine-expressing cells were observed in the recto-sigmoid colon CD4^+^ T cell compartment across all cytokines evaluated. The recto-sigmoid colon CD8^+^ T cell compartment had higher proportions of IFNy-expressing cells, with similar proportions of CD8^+^ T cells expressing the other cytokines in both locations. On the other hand, NK and γδ T cells exhibited a more significant proportion of blood-derived cells expressing IFNγ, and both IFNγ and TNFα, respectively. In this context, as for polyfunctionality, more CD4^+^ T cells from the recto-sigmoid colon produced 2 or more cytokines than those from the blood. Given their cytokine production, the higher expression of PD-1 observed in RMC, compared to peripherical blood, is likely not a sign of exhaustion. This enhanced PD-1 expression observed in the RMC may be more indicative of their memory phenotype. Like the CD4^+^ T cells, more NK and γδ T cells from the blood produced 2 or more cytokines. To our knowledge, this is the first report documenting the cytokine production by a wide variety of T cells from the gut of healthy individuals.

Our data provide immunological insights into the expression of cellular markers of interest in healthy individuals. The findings described here can be used as a tool for understanding a plethora of diseases where gut-derived cells have been shown to play a role in pathogenesis. For instance, the results for CCR6, being both a gut and brain homing marker, highlight a potential gut-link to a typically CNS-centric disease, as elevated levels of CCR6^+^ cells have been detected in the CSF of MS patients (8, 9). Indeed, in MS, the bidirectional crosstalk between the gut and CNS has been extensively investigated, and enhanced intestinal permeability has been observed in patients with MS (39–41). α4β7 is of interest in HIV infection, another disease known for its impact on the gut (10, 14, 15, 26), and in IBD (ie. Crohn’s disease and ulcerative colitis), where antibodies against various integrin combinations [α4, β7, α4β7 (vedolizumab)] have been developed as therapeutic agents [reviewed in (13)]. Beyond diseases where gut-derived cells have been implicated, blockage of CCR5 expression has been evaluated as a treatment in cases where it is used as a co-receptor by a pathogen (HIV, Staphylococcus aureus) or a potential driver of aberrant inflammation (rheumatoid arthritis, IBD, MS, cancer) (21, 41). Together, these data can potentially be used to guide the development of new, targeted, drugs and the possible identification of molecules that could be used as biomarkers for disease development or progression.

The similarity between our results and studies involving surgically obtained tissue explants identifies this colon biopsy method as a viable and less invasive approach to studying the immune profile of at least one component of the gastrointestinal tract. As others have shown that cell proportions and their phenotype can differ by exact location, it highlights the importance of a reproducible sampling method. While the precise tissue composition of the pinch biopsies is not determined (proportion of epithelial, mucosa, lamina propria, lymphoid follicle), collecting several distributed pinches minimizes the potential risk of sampling error and the contribution of sample-to-sample variability.

Since colon biopsies on healthy younger individuals were not readily available, a pitfall of the present study is the difference in age of the donors providing PBMC and RMC. However, studies of T cell composition in the gastrointestinal tract (isolated lymphoid follicles (ILF) anatomy, T cell density in ILFs frequencies of CD4^+^ and CD8^+^ T cells in Peyer’s patches, and jejunal and colonic ILFs etc.) have shown little difference between those of middle (24-49 years) and of older (50+ years) age (35). Additionally, Dock and colleagues reported that, when comparing CD8^+^ T cells from the blood and the gut, the age-related phenotypic differences observed in the gut were minor and limited to memory phenotype (31). These results suggest that the differences between peripheral blood and gut are not likely due to differences in age.

Digestion of gut samples is required for optimal immune cell isolation, providing significantly greater lymphocyte yield compared to mechanical techniques alone (42). It is worth noting, however, that this has a potential impact on the expression level of some epitopes. Our digestion protocol avoids an epithelium removal step, a process which has been shown to impact expression of different surface markers, including α4β7 (43). In a study with human gut T cells, while CXCR5 was shown to be highly susceptible to collagenase IV digestion, it did not appear to have an impact on CD3, CD4, CD8, CD45RA, CD103 and PD-1 expression (44).

Together, our results give a broad picture of the phenotype and function of T and NK cells in the gut and how they compare to cells within the circulation. Additionally, our study demonstrates that biopsies collected during routine colonoscopies can be used as an approach to longitudinal evaluation of gut-derived immune cells in clinical settings, something not realistic or feasible with the use of surgical explants. This type of investigation is particularly important as it describes the cell populations expected in the healthy condition and can be used for determining, among other potential players (e.g. microbiota composition), markers that could indicate important cellular changes particularly in scenarios where gut-derived immune cells are implicated in immunopathogenesis.

## Data availability statement

The original contributions presented in the study are included in the article/supplementary materials, further inquiries can be directed to the corresponding author/s.

## Ethics statement

The studies involving humans were approved by Ottawa Hospital Research Institute Ethics Board (OHRI REB 2005256-01H) (Ottawa, ON, Canada). The studies were conducted in accordance with the local legislation and institutional requirements. The participants provided their written informed consent to participate in this study.

## Author contributions

SCB: Conceptualization, Methodology, Validation, Writing – review & editing, Formal analysis, Investigation, Writing – original draft, Data curation. PB: Conceptualization, Formal analysis, Investigation, Methodology, Validation, Writing – original draft, Writing – review & editing. TB: Data curation, Investigation, Methodology, Validation, Writing – review & editing. SNB: Methodology, Writing – review & editing. MM: Writing – review & editing, Conceptualization. DWC: Writing – review & editing, Funding acquisition, Resources. JA: Conceptualization, Funding acquisition, Methodology, Resources, Supervision, Validation, Writing – review & editing.

## References

[1] Peters A Wekerle H. Autoimmune diabetes mellitus and the leaky gut. Proc Natl Acad Sci USA (2019) 116:14788–90. doi: 10.1073/pnas.1909224116 PMC6660743 31289225

[2] Sorini C Cosorich I Lo CM De Giorgi L Facciotti F Lucianò R. Loss of gut barrier integrity triggers activation of islet-reactive T cells and autoimmune diabetes. Proc Natl Acad Sci USA (2019) 116:15140–9. doi: 10.1073/pnas.1814558116 PMC6660755 31182588

[3] Rao R. Endotoxemia and gut barrier dysfunction in alcoholic liver disease. Hepatology (2009) 50:638–44. doi: 10.1002/hep.23009 PMC6209509 19575462

[4] Riva A Patel V Kurioka A Jeffery HC Wright G Tarff S. Mucosa-associated invariant T cells link intestinal immunity with antibacterial immune defects in alcoholic liver disease. Gut (2018) 67:918–30. doi: 10.1136/gutjnl-2017-314458 PMC5890654 29097439

[5] Merlini E Cerrone M van Wilgenburg B Swadling L Stefania Cannizzo E Monforte AD. Association between impaired vα7.2+cd161++cd8+ (MAIT) and vα7.2+cd161-cd8+ t-cell populations and gut dysbiosis in chronically HIV-and/or HCV-infected patients. Front Microbiol (2019) 10:1–16. doi: 10.3389/fmicb.2019.01972 31555223 PMC6722213

[6] Bandera A De Benedetto I Bozzi G Gori A. Altered gut microbiome composition in HIV infection. Curr Opin HIV AIDS (2018) 13:73–80. doi: 10.1097/COH.0000000000000429 29045252

[7] Nowak P Troseid M Avershina E Barqasho B Neogi U Holm K. Gut microbiota diversity predicts immune status in HIV-1 infection. AIDS (2015) 29:2409–18. doi: 10.1097/QAD.0000000000000869 26355675

[8] Buscarinu MC Fornasiero A Romano S Ferraldeschi M Mechelli R Reniè R. The contribution of gut barrier changes to multiple sclerosis pathophysiology. Front Immunol Front Media S.A (2019) 10. doi: 10.3389/fimmu.2019.01916 PMC6724505 31555257

[9] Buscarinu MC Romano S Mechelli R Pizzolato Umeton R Ferraldeschi M Fornasiero A. Intestinal permeability in relapsing-remitting multiple sclerosis. Neurotherapeutics (2018) 15:68–74. doi: 10.1007/s13311-017-0582-3 29119385 PMC5794695

[10] Peachman KK Karasavvas N Chenine AL McLinden R Rerks-Ngarm S Jaranit K. Identification of new regions in HIV-1 gp120 Variable 2 and 3 Loops that Bind to α4β7 Integrin Receptor. PloS One (2015) 10:1–25. doi: 10.1371/journal.pone.0143895 PMC4666614 26625359

[11] Habtezion A Nguyen LP Hadeiba H Butcher EC. Leukocyte trafficking to the small intestine and colon. Gastroenterology (2016) 150:340–54. doi: 10.1053/j.gastro.2015.10.046 PMC4758453 26551552

[12] Cepek KL Shaw SK Parker CM Russell GJ Morrow JS Rimm DL. Adhesion between epithelial cells and T lymphocytes mediated by E-cadherin and the alpha E beta 7 integrin. Nature (1994) 372:190–3. doi: 10.1038/372190a0 7969453

[13] Rivera-Nieves J. Strategies that target leukocyte traffic in inflammatory bowel diseases. Curr Opin Gastroenterol (2015) 31:441–8. doi: 10.1097/MOG.0000000000000218 PMC4654463 26398681

[14] Arthos J Cicala C Martinelli E Macleod K Van Ryk D Wei D. HIV-1 envelope protein binds to and signals through integrin α4β7, the gut mucosal homing receptor for peripheral T cells. Nat Immunol (2008) 9:301–9. doi: 10.1038/ni1566 18264102

[15] Byrareddy SN Arthos J Cicala C Villinger F Ortiz KT Little D. Sustained virologic control in SIV+ macaques after antiretroviral and 4 7 antibody therapy. Sci (1979) (2016) 354:197–202. doi: 10.1126/science.aag1276 PMC5405455 27738167

[16] Wang C Kang SG Lee J Sun Z Kim CH. The roles of CCR6 in migration of Th17 cells and regulation of effector T-cell balance in the gut. Mucosal Immunol (2009) 2:173–83. doi: 10.1038/mi.2008.84 PMC2709747 19129757

[17] Arthos J Cicala C Nawaz F Byrareddy SN Villinger F Santangelo PJ. The role of integrin α4β7 in HIV pathogenesis and treatment. Curr HIV/AIDS Rep (2018) 15:127–35. doi: 10.1007/s11904-018-0382-3 PMC5882766 29478152

[18] Ansari AA Byrareddy SN. The role of integrin expressing cells in modulating disease susceptibility and progression (January 2016). Int Trends Immun [Internet] (2016) 4:11–27. 28770236 PMC5536173

[19] Fenton TM Jørgensen PB Niss K Rubin SJS Mörbe UM Riis LB. Immune profiling of human gut-associated lymphoid tissue identifies a role for isolated lymphoid follicles in priming of region-specific immunity. Immunity (2020) 52:557–70. doi: 10.1016/j.immuni.2020.02.001 PMC7155934 32160523

[20] Ickler J Francois S Widera M Santiago ML Dittmer U Sutter K. HIV infection does not alter interferon α/β receptor 2 expression on mucosal immune cells. PloS One (2020) 15:1–18. doi: 10.1371/journal.pone.0218905 PMC6959566 31935222

[21] Vangelista L Vento S. The expanding therapeutic perspective of CCR5 blockade. Front Immunol (2018) 8:1981. doi: 10.3389/fimmu.2017.01981 29375583 PMC5770570

[22] Gosselin A Monteiro P Chomont N Diaz-Griffero F Said EA Fonseca S. Peripheral blood CCR4 + CCR6 + and CXCR3 + CCR6 + CD4 + T cells are highly permissive to HIV-1 infection. J Immunol (2010) 184:1604–16. doi: 10.4049/jimmunol.0903058 PMC4321756 20042588

[23] Reboldi A Coisne C Baumjohann D Benvenuto F Bottinelli D Lira S. C-C chemokine receptor 6–regulated entry of TH-17 cells into the CNS through the choroid plexus is required for the initiation of EAE. Nat Immunol (2009) 10:514–23. doi: 10.1038/ni.1716 19305396

[24] Wolburg H Paulus W. horoid plexus: biology and pathology. Acta Neuropathol (2010) 119:75–88. doi: 10.1007/s00401-009-0627-8 20033190

[25] Yamazaki T Yang XO Chung Y Fukunaga A Nurieva R Pappu B. CCR6 regulates the migration of inflammatory and regulatory T cells. J Immunol (2008) 181:8391–401. doi: 10.4049/jimmunol.181.12.8391 PMC2752441 19050256

[26] Cicala C Arthos J Fauci AS. HIV-1 envelope, integrins and co-receptor use in mucosal transmission of HIV. J Transl Med (2010) 9:S2. doi: 10.1186/1479-5876-9-S1-S2 PMC3105502 21284901

[27] Linderman GC Rachh M Hoskins JG Steinerberger S Kluger Y. Fast interpolation-based t-SNE for improved visualization of single-cell RNA-seq data. Nat Methods (2019) 16:243–5. doi: 10.1038/s41592-018-0308-4 PMC6402590 30742040

[28] Van Gassen S Callebaut B Van Helden MJ Lambrecht BN Demeester P Dhaene T. FlowSOM: Using self-organizing maps for visualization and interpretation of cytometry data. Cytometry Part A (2015) 87:636–45. doi: 10.1002/cyto.a.22625 25573116

[29] Schönrich G Raftery MJ. The PD-1/PD-L1 axis and virus infections: A delicate balance. Front Cell Infect Microbiol (2019) 9. doi: 10.3389/fcimb.2019.00207 PMC6584848 31263684

[30] Schieferdecker HL Ullrich R Hirseland H Zeitz M. T cell differentiation antigens on lymphocytes in the human intestinal lamina propria. J Immunol [Internet] (1992) 149:2816–22. doi: 10.4049/jimmunol.149.8.2816 1383328

[31] Dock J Ramirez CM Hultin L Hausner MA Hultin P Elliott J. Distinct aging profiles of CD8+ T cells in blood versus gastrointestinal mucosal compartments. PloS One (2017) 12:1–21. doi: 10.1371/journal.pone.0182498 PMC5568404 28832609

[32] Ribot JC Lopes N Silva-Santos B. γδ T cells in tissue physiology and surveillance. Nat Rev Immunol (2020) 10:221–32. 10.1038/s41577-020-00452-4 33057185

[33] Poggi A Benelli R Venè R Costa D Ferrari N Tosetti F. Human gut-associated natural killer cells in health and disease. Front Immunol (2019) 10:1–18. doi: 10.3389/fimmu.2019.00961 31130953 PMC6509241

[34] Mörbe UM Jørgensen PB Fenton TM von Burg N Riis LB Spencer J. Human gut-associated lymphoid tissues (GALT); diversity, structure, and function. Mucosal Immunol (2021) 14:793–802. doi: 10.1038/s41385-021-00389-4 33753873

[35] Senda T Dogra P Granot T Furuhashi K Snyder ME Carpenter DJ. Microanatomical dissection of human intestinal T-cell immunity reveals site-specific changes in gut-associated lymphoid tissues over life. Mucosal Immunol (2019) 12:378–89. doi: 10.1038/s41385-018-0110-8 PMC6375790 30523311

[36] Bottois H Ngollo M Hammoudi N Courau T Bonnereau J Chardiny V. KLRG1 and CD103 expressions define distinct intestinal tissue-resident memory CD8 T cell subsets modulated in crohn’s disease. Front Immunol (2020) 11:1–13. doi: 10.3389/fimmu.2020.00896 32477365 PMC7235448

[37] FitzPatrick MEB Provine NM Garner LC Powell K Amini A Irwin SL. Human intestinal tissue-resident memory T cells comprise transcriptionally and functionally distinct subsets. Cell Rep (2021) 34:108661. doi: 10.1016/j.celrep.2020.108661 33472060 PMC7816164

[38] Calenda G Keawvichit R Arrode-Brusés G Pattanapanyasat K Frank I Byrareddy SN. Integrin α4β7 blockade preferentially impacts CCR6+ Lymphocyte subsets in blood and mucosal tissues of naive rhesus macaques. J Immunol (2018) 200:810–20. doi: 10.4049/jimmunol.1701150 PMC5760460 29196458

[39] Wong MT Ong DEH Lim FSH Teng KWW McGovern N Narayanan S. A high-dimensional atlas of human T cell diversity reveals tissue-specific trafficking and cytokine signatures. Immunity (2016) 45:442–56. doi: 10.1016/j.immuni.2016.07.007 27521270

[40] Kumar BV Ma W Miron M Granot T Guyer RS Carpenter DJ. Human tissue-resident memory T cells are defined by core transcriptional and functional signatures in lymphoid and mucosal sites. Cell Rep (2017) 20:2921–34. doi: 10.1016/j.celrep.2017.08.078 PMC5646692 28930685

[41] Pervaiz A Ansari S Berger MR Adwan H. CCR5 blockage by maraviroc induces cytotoxic and apoptotic effects in colorectal cancer cells. Med Oncol (2015) 32:1–10. doi: 10.1007/s12032-015-0607-x 25840792

[42] Schreurs RRCE Drewniak A Bakx R Corpeleijn WE Geijtenbeek THB van Goudoever JB. Quantitative comparison of human intestinal mononuclear leukocyte isolation techniques for flow cytometric analyses. J Immunol Methods (2017) 445:45–52. doi: 10.1016/j.jim.2017.03.006 28274838

[43] Van Damme N Baeten D De Vos M Demetter P Elewaut D Mielants H. Chemical agents and enzymes used for the extraction of gut lymphocytes influence flow cytometric detection of T cell surface markers. J Immunol Methods (2000) 236:27–35. doi: 10.1016/S0022-1759(99)00243-4 10699577

[44] Trapecar M Khan S Roan NR Chen TH Telwatte S Deswal M. An optimized and validated method for isolation and characterization of lymphocytes from HIV+ Human gut biopsies. AIDS Res Hum Retroviruses (2017) 33:S31–9. doi: 10.1089/aid.2017.0208 PMC5684666 28882052

